# Outcomes of pars plana vitrectomy following ocular trauma at varying surgical time points

**DOI:** 10.1186/s40942-022-00399-9

**Published:** 2022-07-25

**Authors:** Muhammad Z. Chauhan, Michalis Georgiou, Hytham Al-Hindi, Sami H. Uwaydat

**Affiliations:** 1 Jones Eye Institute, University of Arkansas for Medical Sciences, 4301 W. Markham St, Little Rock, AR 72205 USA; 2 Miami Integrative Metabolomics Research Center, Bascom Palmer Eye Institute, University of Miami, Miami, FL USA; 3 Moorfields Eye Hospital, London, UK

**Keywords:** Trauma, Pars plana vitrectomy, Timing, Outcomes, Ocular injury

## Abstract

**Background:**

The optimal timing of pars plana vitrectomy (PPV) following ocular trauma is an ongoing debate. Early vitrectomy post-trauma enables the rapid assessment of retinal disease by removing the scaffold that fosters proliferative vitreoretinopathy. On the other hand, late vitrectomy is less challenging as there is a lower risk of bleeding and posterior vitreous detachment induction is easier. The purpose of this work is to report the functional and anatomical outcomes following ocular traumatic injuries in a United States-based cohort, emphasizing the time of intervention.

**Methods:**

This was a retrospective case series of 110 patients with traumatic ocular injuries who underwent PPV between 2008 to 2020. Patients were grouped into four timing categories: same day (0 days), early (1–7 days), delayed (8–14 days), and late (> 14 days). Multivariable regression models controlling for confounding were implemented to assess the impact of vitrectomy timing on anatomical and functional outcomes. Visual acuity (VA) at baseline and after surgery, proliferative vitreoretinopathy (PVR), and enucleation for each vitrectomy timing category were recorded.

**Results:**

Patient demographics and severity of ocular trauma were comparable across timing categories. Final VA in LogMAR was found to have a stepwise worsening as the time of ocular trauma to vitrectomy was increased (p < 0.05). For every one-step increase in the vitrectomy timing category, there was an adjusted 0.24 (CI 0.04–0.44) increase in final VA. No patient in the same day vitrectomy group had an enucleation or PVR, while patients who had late vitrectomies had the largest number of both enucleations and PVR (44.4% and 52.0%, respectively). In adjusted analysis, there was 3.11 increased odds (CI 1.03–9.42) of developing PVR for a one-step increase in vitrectomy timing (p < 0.05).

**Conclusion:**

Vitrectomy on the same day of injury has the best final VA, and the lowest incidence rates of PVR and enucleation in comparison to other timing categories, regardless of etiology.

**Supplementary Information:**

The online version contains supplementary material available at 10.1186/s40942-022-00399-9.

## Introduction

It is estimated that there are around 2.0 million cases of eye trauma that occur each year in the United States, with approximately 1 million individuals having permanent visual impairment because of injury [1]. Pars plana vitrectomy (PPV) has played a major role in improving outcomes of ocular trauma by allowing posterior segment reconstruction, clearing vitreous opacities, and preventing endophthalmitis [2]. However, the optimum timing of PPV has been a topic of on-going debate [3–5]. Early vitrectomy allows for prompt evaluation and treatment of retinal pathology, with the removal of the scaffold that promotes proliferative vitreoretinopathy (PVR). However, this can be particularly challenging due to excessive bleeding. Late vitrectomy, on the other hand, is less challenging as the risk of bleeding is lower and the induction of a posterior vitreous detachment (PVD) is easier. Treatment parameters of trauma are based on results of vitrectomy performed decades ago with more rudimentary equipment and poor visualization modalities. In the past, the wait time was deemed necessary to improve visibility. For example, performing a vitrectomy with 20 g and no valved cannulas was very cumbersome and difficult in traumatized eyes. This, however, is not the case with 23 g vitrectomy with valved cannulas and intraocular pressure control. Reassessment of older notions given the new realities and technological advances available at present are needed. Our study aimed to investigate the impact of early PPV on functional and anatomical outcomes following open and closed-globe injuries. Functional outcomes were assessed by final VA, while anatomical outcomes were assessed primarily according to enucleation and presence of PVR at varying PPV time points.

## Methods

The study protocol was approved by the Institutional Review Board of the University of Arkansas for the Medical Sciences (UAMS) and was performed in accordance with the Declaration of Helsinki. A retrospective chart review was performed of all patients with globe injuries that were hospitalized at the Jones Eye Institute at UAMS, and who underwent initial repair or exploration and subsequent PPV between 2008 and 2020. A single surgeon performed all initial PPVs, although multiple surgeons were involved in the primary wound closure or globe exploration. All data were anonymized and de-identified before analysis. Indications for vitrectomy following ocular trauma included [1] intraocular foreign bodies, [2] suspected posterior capsule rupture, [3] dense vitreous hemorrhage with b scan suspicious for retinal detachment, [4] zone 2/3 injuries (zone 1 may also have been involved), and/or [5] endophthalmitis. All vitrectomies were performed using the 23 g Constellation system (Alcon Laboratories Inc, USA). The patients’ charts found not to have documented initial visual acuity were excluded from statistical analysis. The ocular trauma score (OTS) was also calculated based on the following variables: globe rupture, endophthalmitis, initial VA, globe perforation, retinal detachment, and the presence of relative afferent pupillary defect. The OTS scores the severity of ocular trauma from a range of 1 to 5, indicating most severe to least severe, respectively. Other data elements collected were timing of initial globe closure (primary intervention), timing to the first PPV from initial injury, best corrected final visual acuity at last follow-up, rate of enucleation, development of PVR, and the type of injury (blunt force, intraocular foreign body (IOFB), sharp injury, and injury from a high-velocity projectile).

We grouped patients into four groups according to the timing of vitrectomy following ocular injury: same day (0 days), early (1–7 days), delayed (8–14 days), and late (> 14 days). Demographic, clinical characteristics, and presenting trauma variables at varying timing categories were compared with chi-square tests for categorical variables and ANOVA for continuous variables. The primary analyses consisted of two regression models. The first was a multiple linear regression that was used to determine the ability of vitrectomy timing categories to predict final VA, while adjusting for injury, gender, zone 3 injury, OTS, and age. The second was a confounder-adjusted logistic regression determining the relationship between vitrectomy timing and the development of PVR. Post-hoc secondary analysis including running regressions on isolated injury types.

In addition, we ran several sensitivity analyses. Sensitivity analyses included running both primary models without IOFB injury type included. We also calculated E-values as a sensitivity analysis to determine the impact of unmeasured confounders in the final regression models. E-values represent the minimum strength of association that a potential unmeasured confounder needs to have to explain the relationship between exposure and outcome [6]. The power of the regression analysis was found to be 0.98 with an *N* = 110 and an alpha of 0.05 while using 6 predictors with an *R*^2^ of at least 0.20. All analyses were performed using Stata Release 14.2 (*StataCorp LP*). Tests with 2-sided P values < 0.05 were considered statistically significant.

## Results

In total, there were 110 patients that presented to our clinic with ocular trauma between 2008 and 2020 and subsequently underwent vitrectomy. Eighty-nine were males (80.91%) and 21 females (19.09%) with an age range of 10–92 years old (mean: 45.2 ± 19.1). Twelve patients had same day, 27 early, 35 delayed, and 36 late vitrectomies with an average length of follow-up of 678 days. We found no statistically significant difference in gender or age between the four vitrectomy timing groups (Table 1). OTS was also not significantly different between the four groups. We found that initial VA, lens status, and endophthalmitis on presentation were not significantly different between timing groups. Of note, there was a statistically significant difference in the probability distributions between the vitrectomy timing groups and injury type, χ^2^(3) = 36.372, p < 0.001. We found that patients who underwent late vitrectomy had higher proportion of blunt force injuries (72.2%), while same-day vitrectomy was associated with a higher proportion of intraocular foreign bodies (75.0%). The proportion of zone 2 injures was also found to be highest in early vitrectomy timing (37.0%) compared to other groups (p < 0.05).

**Table 1:** Demographics and presenting history of patients with same day, early, delayed and late vitrectomy timing

	Same day	Early (1-7 days)	Delayed (8–14 days)	Late (> 14 days)	P-value^*^
Total, No. (%)	12 (10.9)	27 (24.6)	35 (31.8)	36 (32.7)	
Demographics					
Gender, No. (%)					
Women	0 (0)	5 (18.5)	8 (22.6)	8 (22.2)	
Men	12 (100)	22 (81.5)	27 (77.14)	28 (77.8)	0.336
Age, mean (SD)	38.9 (14.8)	44.2 (21.6)	46.1 (19.6)	47.0 (17.9)	0.261
Lens status, No. (%)					
Rupture	8 (66.7)	12 (44.4)	10 (30.3)	6 (26.1)	
Aphakic	0 (0)	3 (11.1)	5 (15.2)	3 (13.0)	
Pseudophakic	(0)	1 (3.70)	0 (0)	1 (4.4)	0.405
Injury type, No. (%)					
Blunt	1 (8.3)	14 (51.9)	23 (65.7)	26 (72.2)	
Sharp	2 (16.7)	7 (25.9)	8 (22.9)	8 (22.2)	
IOFB	9 (75.0)	5 (18.5)	2 (5.56)	2 (5.56)	
Projectile	0 (0)	1 (3.7)	0 (0)	0 (0)	** < 0.001**
Final VA, mean (SD)	1.11 (0.98)	1.40 (0.94)	1.77 (1.0)	1.97 (0.69)	** < 0.05**
Enucleation, No. (%)	0 (0)	2 (22.2)	3 (33.3)	4 (44.4)	0.680
PVR, No. (%)	0 (0)	2 (8.0)	10 (40.0)	13 (52.0)	** < 0.01**
OTS, mean (SD)^a^	2.45 (0.52)	2.31 (0.89)	1.72 (0.92)	2.12 (0.88)	0.589
Initial VA, No. (%)	2.03 (0.76)	1.99 (0.82)	2.33 (0.54)	2.29 (0.41)	0.097
Endophthalmitis, No. (%)	1 (20.0)	2 (40.0)	1(20.0)	1 (20.0)	0.707

*OTS* ocular trauma score, *VA* visual acuity, *IOFB* intraocular foreign body, *PVR* proliferative vitreoretinopathy, *SD* standard deviation, No., frequency

*P-values for χ^2^ test for categorical variables and ANOVA for continuous

^a^OTS score utilizes the variables initial vision, globe rupture, endophthalmitis, perforating injury, retinal detachment, and afferent pupillary defect (APD)

We subsequently examined the functional and anatomical outcomes in each vitrectomy timing category. Initial VA was not significantly different between timing categories. However, in examining final VA at each category, we found a stepwise worsening of final VA for each subsequent group, F(3, 107) = 3.73, p < 0.05. Patients who underwent vitrectomy on the same day as the injury (VA 1.11 ± 0.98 LogMAR) had the best final VA, while patients who had late vitrectomies had the worst final VA (1.97 ± 0.69 LogMAR) (p < 0.05). To control for unmatched differences in demographic characteristics, trauma severity, and injury type, we conducted a multiple linear regression analysis to determine the ability of vitrectomy timing to predict the variability in final vision. There was independence of residuals, as assessed by a Durbin-Watson statistic of 1.65. There was homoscedasticity and normality of the residuals. The regression model was found to explain 33% of the variability in final VA with an adjusted *R*^2^ = 28.0%. Final visual acuity on final follow-up date was statistically significantly predicted by vitrectomy timing (p < 0.05) and ocular trauma severity (p < 0.001). We found that for every one-step increase in vitrectomy timing category, there was a 0.24 LogMAR (CI 0.04–0.44) increase in final VA (worse vision) while adjusting for trauma severity (Table 2). We next evaluated the frequency of PVR and enucleations as a measure of anatomical outcomes. No patients in the same-day vitrectomy group had an enucleation or PVR, while patients who had late vitrectomies had the largest number of both enucleations and PVR (44.4% and 52.0%, respectively) (Table 1). Interestingly, there was a stepwise increase in the frequency of enucleations from same day to late vitrectomy; however, this observation was not statistically significant (p = 0.680). On the other hand, there was a statistically significant stepwise increase in the frequency of PVR from the same day to late (p < 0.01). A logistic regression model controlling for demographic characteristics, trauma severity, and presenting history found that there were 3.11 increased odds (CI 1.03–9.42) of developing PVR for a one-step increase in vitrectomy timing (p < 0.05) (Table 3).

**Table 2:** Multiple regression model for predicting final visual acuity

Final VA	*B*	95% CI for *B*		*SE B*	ß	*R*^2^	∆*R*^2^
		LL	UL				
Model						0.33	0.28
Constant	1.89***	0.91	2.88	0.49			
Vit. timing	0.24*	0.04	0.44	0.09	0.25		
Age	0.004	–0.006	0.013	0.005	0.083		
Gender	–0.28	–0.71	0.15	0.22	–0.11		
OTS	–0.48***	–0.72	–0.24	0.12	–0.45		
Injury type	–0.08	–0.33	0.17	0.13	–0.07		
Zone 3	–0.09	–0.49	0.32	0.20	–0.05		

*B* unstandardized regression coefficient, *CI* confidence interval, *LL* lower limits, *UL* upper limit, *SEB* standard error of the coefficient, *ß* standardized coefficient, *R*^2^ coefficient of determination, ∆*R*^2^ adjusted *R*^2^

^*^p < 0.05, **p < 0.01, ***p < 0.001

**Table 3:** Odds ratios for development of PVR following vitrectomy at varying time points

PVR	Odds ratio	95% CI	P-value
Vitrectomy timing	3.11	1.03–9.42	**0.044**
Type of injury	0.26	0.52–1.31	0.102
Gender	1.12	0.19–6.33	0.895
Zone 3	3.27	0.72–14.89	0.124
OTS	0.14	0.04–0.52	**0.004**
Age	1.02	0.05–1.30	0.476

*PVR* proliferative vitreoretinopathy, TS ocular trauma score

^a^Logistic regression model with PVR as dependent variable adjusting for vitrectomy timing, type of injury, gender, zone 3 injury, OTS, and age

In sensitivity analysis removing IOFB as an injury type, it was found that vitrectomy timing and OTS remained the only two significant confounders associated with the development of PVR and worse final vision (Additional file 1: Tables S1 and S2). E-value sensitivity analysis for unmeasured confounding was calculated for the adjusted regression coefficients and odds ratios from primary regression analysis for final VA and PVR development, respectively. The adjusted regression coefficient point estimate of 0.242 for the final vision associated with the vitrectomy timing category corresponded to an E-value of 1.85, and for the confidence interval value closest to the null, the E-value was 1.27. The adjusted odds ratio point estimate of 3.11 for the development of PVR associated with the vitrectomy timing category corresponded to an E-value of 5.67, and for the confidence interval value closest to the null, 1.03, the E-value was 1.21.

## Discussion

One of the major causes of visual impairment around the globe is ocular trauma. Pars plana vitrectomy has been shown to provide good visual outcomes for patients who undergo vitreoretinal surgery after ocular trauma [7]. Vitrectomy post-trauma aids in providing a clearer visual axis, identifying retinal breaks, preventing tractional retinal detachments, and decreasing rates of PVR [8, 9]. However, PPV is not without complications, including cataract formation, retinal detachment, vitreous hemorrhage, elevated intraocular pressure, and post-operative outer retinal folds [10–12]. Despite the potential complications, the benefits of PPV following ocular trauma outweigh the risks with an overall good prognostic value [13]. Although, the optimal timing of PPV following ocular trauma to maximize these benefits is a heavily debated subject [3–5, 14–21]. Prevalent thought suggests a stepwise approach where the primary wound is closed on day 1 and a vitrectomy is performed during the second week [21]. In reality, vitrectomy timing varies extensively from several days to weeks after the initial insult. Recently published findings from a cross-sectional survey of eye trauma experts found that in cases where vitrectomy was indicated, 45.5% of experts preferred vitrectomy ≥ 7 days after the repair of open globe injuries [12]. A review of the literature shows that the most favorable time to perform vitrectomy after ocular trauma varies significantly from within 3 days [22], within 4 days [14, 17, 18], 4–10 days [23], 8–14 days [20], and even up to 15–30 days [19] post-injury. It should be noted, however, that the inclusion and exclusion criteria also vary across studies. Furthermore, the diverse nature of ocular injuries makes it difficult to create a universal framework for vitrectomy timing. One study sought to accomplish this by creating a flexible vitrectomy timing system to minimize the incidence of PVR [17]. However, this study did not include patients diagnosed with endophthalmitis or IOFB at presentation. IOFBs are often excluded from studies since, in most cases, vitrectomy is typically performed within 48 h to decrease the risk of endophthalmitis and PVR development. Yet, some cases are referred to tertiary care centers leading to natural variation in the timing of IOFB removal. In addition, a review of the literature suggests that there is debate regarding a delayed vs. immediate approach to the removal of IOFBs [24]. Delayed removal is often unavoidable in cases where other procedures, such as neurosurgery, are more pressing. In our study, approximately 11% of IOFBs were removed ≥ 8 days following ocular trauma. To account for any effects of IOFB injuries driving better earlier outcomes, we ran sensitivity analysis by removing IOFB injury type (Additional file 1: Tables S1 and S2). We found that trends remained the same without IOFB as an etiology of ocular trauma.

There are currently three published randomized controlled trials, which included 173 participants in totality, that have examined the optimal vitrectomy timing for open globe injuries [18, 25, 26]. All these studies were conducted in China. He et al. (2020) included 53 patients with open globe injuries and randomized participants into an early vitrectomy group (within 4 days) and a late vitrectomy group (10–14 days post-injury). They found that vitrectomy within 4 days resulted in significantly better best-corrected visual acuity (BCVA) (p = 0.041). Surgery that occurred 10–14 days post-injury was associated with higher rates of PVR (p = 0.054) and lower rates of retinal reattachment (p = 0.001). There was no statistically significant difference in the complication rates between groups. X. Y. Wu (2015) (N = 58) and J. Q. Lu (2016) (N = 62) randomized participants into an early vitrectomy group (within 3 days) and a delayed group (1–2 weeks). They both found the early group had significantly better VA (p < 0.05) and lower post-operative complications (p < 0.05). All three studies came to similar conclusions that earlier is better in terms of better final VA and lower rates of complications. However, across these studies, the method of randomization and masking of participants/researchers was not clear.

Our analysis adds to these studies by showing that early vitrectomy, preferably on the same day of injury, is associated with the best final VA with decreased risk of PVR while controlling for trauma severity and type of injury (e.g., IOFB, blunt, sharp). We found outcomes worsened with a stepwise delay in vitrectomy timing from same day of injury to 1–7 days and then to 8–14 days, with greater than 14 days presenting with the worst overall outcomes. We also found a stepwise increase (non-significant) in the frequency of enucleations from same day to late vitrectomy However, despite these findings that earlier is better, variability of patient presentation and organizational infrastructure may dictate the optimal timing for certain cases. The timing of surgery hinges on multiple organizational factors including the availability of OR time, issues with doing off-hours cases, and surgeon availability. Findings from this work suggest that PPV as soon as reasonably possible following ocular trauma, regardless of injury type and severity, appears to be beneficial.

A strength and a limitation of this work includes the use of data from a single academic institution. This is a strength in that this analysis controls for inter-surgeon variability. However, this is a limitation due to the potentially decreased variation in patient presentation. Despite our study being one of the larger studies examining optimal vitrectomy timing, the sample size is still limited. Given the retrospective nature of this study, selection bias is a potential concern. However, to mitigate bias we utilized the OTS as a calculation for trauma severity and ran sensitivity analyses removing IOFB as an injury type. We found that trauma severity was comparable across timing groups. Randomized trials with longer follow-up periods, larger sample sizes, and focused endpoints by injury etiology can further investigate the evidence provided herein. The outcome data consisted of VA at the most recent follow-up assessment. Interval-based VA outcome analysis was not performed. This work provides evidence that vitrectomy on the same day of injury, has the best final VA and the lowest rates of PVR, regardless of etiology. These findings support that early vitrectomy provides better functional and anatomical outcomes. Further evaluation on the optimal time of intervention after ocular trauma may be warranted to determine the additional effects of delayed surgery.

## Supplementary Information

**Additional file 1: Table S1.** Sensitivity analysis removing IOFB in multiple regression model for predicting final visual acuity. **Table S2.** Sensitivity analysis removing IOFB of odds ratios for development of PVR following vitrectomy at varying time points.

## Data Availability

The datasets generated during and/or analyzed during the current study are available from the corresponding author on reasonable request.

## Abbreviations

PVR: Proliferative vitreoretinopathy
PPV: Pars plana vitrectomy
PVD: Posterior vitreous detachment
OTS: Ocular trauma score
VA: Visual acuity
IOFB: Intraocular foreign body

## References

[1] Iftikhar M, Latif A, Farid UZ, Usmani B, Canner JK, Shah SMA (2019). Changes in the incidence of eye trauma hospitalizations in the United States from 2001 through 2014. JAMA Ophthalmol.

[2] Mansouri MR, Tabatabaei SA, Soleimani M, Kiarudi MY, Molaei S, Rouzbahani M (2016). Ocular trauma treated with pars plana vitrectomy: early outcome report. Int J Ophthalmol.

[3] Kuhn F (2014). The timing of reconstruction in severe mechanical trauma. Ophthalmic Res.

[4] Ghoraba HH, Heikal MA, Mansour HO, Abdelfattah HM, Elgemai EM, Zaky AG (2016). Timing of pars plana vitrectomy in management of gunshot perforating eye injury: observational study. J Ophthalmol.

[5] Agrawal R, Shah M, Mireskandari K, Yong GK (2013). Controversies in ocular trauma classification and management: review. Int Ophthalmol.

[6] Haneuse S, VanderWeele TJ, Arterburn D (2019). Using the E-value to assess the potential effect of unmeasured confounding in observational studies. JAMA.

[7] Ung C, Stryjewski TP, Eliott D (2020). Indications, findings, and outcomes of pars plana vitrectomy after open globe injury. Ophthalmol Retina.

[8] Aziz M, Patel S (2018). BB gun-related open globe injuries. Ophthalmol Retina.

[9] Aylward GW (2014). 25th RCOphth congress, president’s session paper: 25 years of progress in vitreoretinal surgery. Eye.

[10] Recchia FM, Ruby AJ, Carvalho Recchia CA (2005). Pars plana vitrectomy with removal of the internal limiting membrane in the treatment of persistent diabetic macular edema. Am J Ophthalmol.

[11] Lee WW, Bansal A, Sadda S, Sarraf D, Berger AR, Wong DT, et al. Outer retinal folds following pars plana vitrectomy vs pneumatic retinopexy for retinal detachment repair: post Hoc analysis from PIVOT. Ophthalmol Retina. 2022 Mar;6(3):234–242. 10.1016/j.oret.2021.09.001 34520841

[12] Xu YX, Liu LP, Li JB, Cheng HH, Hou M, Lu L (2021). Vitreoretinal surgeons' experience and time interval from pars-plana vitrectomy to cataract extraction. Int J Ophthalmol.

[13] Fujikawa A, Mohamed YH, Kinoshita H, Matsumoto M, Uematsu M, Tsuiki E (2018). Visual outcomes and prognostic factors in open-globe injuries. BMC Ophthalmol.

[14] Akincioglu D, Kucukevcilioglu M, Durukan AH (2021). Pars plana vitrectomy timing in deadly weapon-related open-globe injuries. Eye.

[15] Dalma-Weiszhausz J, Quiroz-Mercado H, Morales-Canton V, Oliver-Fernandez K, De Anda-Turati M (1996). Vitrectomy for ocular trauma: a question of timing?. Eur J Ophthalmol.

[16] de Juan E, Sternberg P, Michels RG (1984). Timing of vitrectomy after penetrating ocular injuries. Ophthalmology.

[17] Han L, Jia J, Fan Y, Yang L, Yue Z, Zhang W (2019). The vitrectomy timing individualization system for ocular trauma (VTISOT). Sci Rep.

[18] He Y, Zhang L, Wang F, Zhu M, Wang Y, Liu Y (2020). Timing influence on outcomes of vitrectomy for open-globe injury: a prospective randomized comparative study. Retina.

[19] Mieler WF, Mittra RA (1997). The role and timing of pars plana vitrectomy in penetrating ocular trauma. Arch Ophthalmol.

[20] Yu H, Li J, Yu Y, Li G, Li D, Guan M (2019). Optimal timing of vitrectomy for severe mechanical ocular trauma: a retrospective observational study. Sci Rep.

[21] Kuhn F, Morris R (2021). Early vitrectomy for severe eye injuries. Eye.

[22] Coles WH, Haik GM (1972). Vitrectomy in intraocular trauma Its rationale and its indications and limitations. Arch Ophthalmol.

[23] Gregor Z, Ryan SJ (1983). Complete and core vitrectomies in the treatment of experimental posterior penetrating eye injury in the rhesus monkey I clinical features. Arch Ophthalmol.

[24] Justin GA, Baker KM, Brooks DI, Ryan DS, Weichel ED, Colyer MH (2018). Intraocular foreign body trauma in operation Iraqi freedom and operation enduring freedom: 2001 to 2011. Ophthalmology.

[25] Lu Junqi. Timing of vitreous surgery in patients with open ocular trauma. International Journal of Ophthalmology 2016;16(9):1765–1767.

[26] Wu XY (2015). Relationship between the timing of vitreous surgery and prognosis after the open globe injuries. Int Eye Sci.

