# Supplementary material for: Outcomes of pars plana vitrectomy following ocular trauma at varying surgical time points
Source: Int J Retina Vitreous. 2022 Jul 25;8:49. doi: 10.1186/s40942-022-00399-9 (PMC9310478; doi:10.1186/s40942-022-00399-9)
Supplement: Supplementary file 1 — Additional file 1: Table S1. Sensitivity analysis removing IOFB in multiple regression model for predicting final visual acuity. Table S2. Sensitivity analysis removing IOFB of odds ratios for development of PVR following vitrectomy at varying time points. [file 40942_2022_399_MOESM1_ESM.docx]

| **Supplemental Table 1.** Sensitivity Analysis Removing IOFB in Multiple Regression Model for Predicting Final Visual Acuity. | | | | | | | |
| --- | --- | --- | --- | --- | --- | --- | --- |
| **Final VA** | ***B*** | **95% CI for *B*** | | ***SE B*** | **ß** | ***R*^2^** | **∆*R*^2^** |
|  |  | **LL** | **UL** |  |  |  |  |
| **Model** |  |  |  |  |  | 0.36 | 0.31 |
| Constant | 1.19*** | 0.86 | 2.96 | 0.52 |  |  |  |
| Vit. Timing | 0.31** | 0.08 | 0.54 | 0.11 | 0.27 |  |  |
| Age | 0.002 | -0.007 | 0.013 | 0.005 | 0.06 |  |  |
| Gender | -0.27 | -0.70 | 0.17 | 0.22 | -0.12 |  |  |
| OTS | -0.56*** | -0.82 | -0.29 | 0.13 | -0.49 |  |  |
| Injury Type | -0.17 | -0.52 | 0.18 | 0.18 | -0.11 |  |  |
| Zone 3 | -0.16 | -0.59 | 0.27 | 0.22 | -0.08 |  |  |
| *Note.* *B,* unstandardized regression coefficient; CI, confidence interval; LL, lower limits; UL, upper limit; *SE B*, standard error of the coefficient; ß, standardized coefficient, *R*^2^, coefficient of determination; ∆*R*^2^, adjusted *R*^2^.  *p <. 05, **p < .01, ***p < .001. | | | | | | | |

| **Supplemental Table 2.** Sensitivity Analysis Removing IOFB of Odds Ratios for Development of PVR Following Vitrectomy at Varying Time Points. | | |  |
| --- | --- | --- | --- |
| **PVR** | **Odds Ratio** | **95% CI** | |
| Vitrectomy Timing | 3.99 | **1.14 - 14.02** | |
| Type of Injury | 0.80 | 0.15 - 4.23 | |
| Gender | 0.77 | 0.12 - 4.89 | |
| Zone 3 | 3.12 | 0.65 - 14.74 | |
| OTS | 0.08 | **0.01 - 0.41** | |
| Age | 1.02 | 0.97 - 1.06 | |
| *Note.* PVR, proliferative vitreoretinopathy; OTS ocular trauma score. * P-values for χ^2^ test for categorical variables and ANOVA for continuous. ^a^ Logistic regression model with PVR as dependent variable adjusting for vitrectomy timing, type of injury (-IOFB), gender, zone 3 injury, OTS, and age. | | |  |
